# The problem with reproductive freedom. Procreation beyond procreators’ interests

**DOI:** 10.1007/s11019-019-09917-3

**Published:** 2019-08-14

**Authors:** Giulia Cavaliere

**Affiliations:** Department of Global Health & Social Medicine, School of Global Affairs, King’s College London, Room 3.12, Bush House NE Wing, 40 Aldwych, London, WC2B 4BG UK

**Keywords:** Procreation, Reproductive freedom, Eugenics, Climate change

## Abstract

Reproductive freedom plays a pivotal role in debates on the ethics of procreation. This moral principle protects people’s interests in procreative matters and allows them discretion over whether to have children, the number of children they have and, to a certain extent, the type of children they have. Reproductive freedom’s theoretical and political emphasis on people’s autonomy and well-being is grounded in an individual-centred framework for discussing the ethics of procreation. It protects procreators’ interests and significantly reduces the permissible grounds for interference by third parties. In this article I show that procreative decisions have far-reaching effects on the composition and size of the population. The upshot of considering these effects allows for the appreciation of the inadequacy of a framework that solely considers individual (i.e. procreators’) interests to discuss the ethics of procreation. To address such inadequacy, I assess costs and benefits of past and present proposals to reflect on procreation in such a way as to consider its far-reaching effects. I conclude by arguing that reproductive freedom should be defended as an imperfect but instrumentally necessary tool. This framing would enable those participating in debates on the ethics of procreative decisions to work towards an ethical framework that accounts for the cumulative effects of these decisions.

## Introduction

Reproductive freedom plays a pivotal role in debates on the ethics of procreation. This moral principle protects people’s interests in procreative matters and allows them discretion over whether or not to have children, the number of children they have and, to a certain extent, the type of children they have (Buchanan et al. 2001; Brock 2005; Harris 1998; Robertson 1994). It is invoked within debates on reproductive technologies, abortion and contraception to defend these practices against restrictions, statutory bans and other kinds of interferences from third parties. Reproductive freedom is at the core of these defences as exercising freedom in procreative matters is thought to be relevant for people’s autonomy and well-being (Brock 2005). According to defenders of such freedom, having and raising children are core human activities that, for many, intersect with dearly held values and notions of a good life (Brock 2005). These activities are also “central to personal identity, to dignity, and to the meaning of one’s life” (Robertson 1994, p. 24).

The recognition of the relevance of exercising freedom in procreative matters for people’s personal autonomy and well-being counts among the theoretical successes of reproductive freedom. In addition, reproductive freedom plays, and has played, an important performative role in contemporary and past political struggles to secure access to contraception, termination of pregnancies, and to allow new assisted reproductive technologies (Cavaliere and Harris 2018). On practical grounds, then, reproductive freedom has a positive track record in terms of protecting people’s interests.

Despite this, defences of reproductive freedom have not gone unchallenged. Conceptual and normative critiques of this principle are manifold.^1^ Some of these critiques are motivated by a broader dissatisfaction with the dominant individualistic understanding of the principle of autonomy within healthcare settings (Dove et al. 2017). Others are motivated by the limited reach of reproductive freedom and its centring around non-interference clauses (Mills 2013). According to this critique, reproductive freedom is narrowly focused on removing statutory bans and other barriers to exercise reproductive rights, while leaving unchallenged structural injustices and other legal, economic, social and political barriers to access services (Roberts 1997). Another set of critiques have targeted the language of ‘choice’ and how it masks the constraints women experience in real-life situations (Lippman 1999; Löwy 2015; Roberts 1997; Rothman 1985; Samerski 2009). According to these critiques, the focus on choice favours commercial, rather than women’s, interests and it renders hegemonic norms and ways of conduct, thereby constraining women’s freedom  (Rothman 1985; Samerski 2009). While these critiques raise important objections, in this article I argue that one of the most problematic features of reproductive freedom concerns the individual-centred framework that this principle rests upon. Reproductive freedom’s theoretical and political emphasis on people’s autonomy, dignity and well-being is conceptually and normatively problematic, as it fails to account for other relevant interests than those of the procreators and for the effects of procreative decisions on these interests.

In what follows, I first illustrate how reproductive freedom is grounded in an individual-centred framework. Then, I provide examples to show that procreative decisions have far-reaching effects that cannot be adequately captured and normatively appraised within such individual-centred framework. To counter the tendency to frame procreative decisions as a private matter, I move to an analysis and evaluation of past and contemporary proposals that adopt a broader framework to reflect on the far-reaching effects of procreative decisions. I conclude by presenting some of the challenges that need to be addressed prior to adopt a broader framework, one that enables to assess normatively desirable and undesirable courses of actions.

## Reproductive freedom and the individual-centred framework

Reproductive freedom protects people’s interests and it significantly reduces the ethically and legally permissible grounds for interference by third parties. Within discourses on reproductive freedom, procreation is often conceived as a private matter and is defended from third parties’ interferences for this reason. This feature of reproductive freedom reflects contemporary Western democracies’ emphasis on protecting people’s freedom of agency from third parties’ interference. Such freedom has its root in John Stuart Mill’s (1979/1859) so called ‘harm principle’, which states that:

[T]he only purpose for which power can be rightfully exercised over any member of a civilized community, against his will, is to prevent harm to others. His own good, either physical or moral, is not a sufficient warrant. (1979/1859)

This Millian presumption in favour of freedom considers the risk of harm occurrence or the occurrence of harm to others the only justifiable grounds for interference and it places the burden of proof on attempts to limit such freedom. As with other freedoms protected in liberal democratic societies, the freedom to decide in matters of procreation is not absolute and other considerations can rightfully constrain its scope (Brock 2005; Dworkin 1993). Such considerations often have a bearing on limits to reproductive freedom put in place to protect the well-being and the interests of the child who is born as a result of the procreative act. For instance, reproductive freedom protects women from interference in the choice of whether to become pregnant and to continue a pregnancy, but it would not protect them from interference against consuming harmful substances during pregnancy. Consuming substances may harm the (future) child and hence constitute a legitimate reason for third-party interference. Other than considerations pertaining to the welfare of the (future) child, interference in reproductive decisions may be justified by concerns for (social) justice (Ross 2006); solidarity (see for instance discussions on genome editing and human reproduction: Nuffield Council 2018); and resource allocation (Rulli 2016a), among others.^2^ Within discourses on reproductive freedom, these and other concerns motivated by protecting the well-being and interests of others are arguably more controversial than those focusing on the welfare of the (future) child. Curtailing or limiting reproductive freedom is often resisted due to the negative impact on people’s well-being and autonomy. As a result, third parties’ interests–as opposed to direct or indirect harms to these third parties–tend to be regarded as less morally significant than the interests of the procreators (Harris 1998; Mills 2013; Robertson 1994).

### The problem with reproductive freedom: procreation and effects on third parties

Despite reproductive freedom’s emphasis on protecting individuals’ (i.e. procreators’) interests, procreative decisions have effects on third parties whose interference reproductive freedom protects against. For instance, these decisions inevitably affect the size and the structure^3^ of the population, which in turn have effects on third parties’ lives and interests. That procreative decisions affect the size of the population seems relatively uncontroversial: some people have siblings, and some do not, due to the procreative behaviours of their parents. This, at the micro-level, affects the lives of these people and the lives of their kin group. Moving to the macro-level, the decline in fertility rates of people living in developed countries and, to a lesser extent, in developing countries, affects the number of people who will inhabit our planet in the future. A trend that depends on the cumulative effects of the procreative decisions of people currently living in these countries. This decline in fertility changes the size of the (future) population and affects people’s lives by, for instance, affecting the economic growth of a given country, its pension schemes, the organisation of its labour market and so forth.^4^

Procreation can also affect the structure of the population. The Zika virus epidemic that began in early 2015 in Latin America provides an example of this. Procreating during the epidemic meant that children had a higher risk of being born with physical abnormalities and developmental disorders than, say, before or after the epidemic. Something similar can be said about hereditable genetic conditions. For known carriers of harmful genetic mutations, procreating ‘naturally’, i.e. without turning to IVF coupled with pre-implantation genetic diagnosis (PGD), means that children born as a result of the procreative act have a higher than normal chance of inheriting harmful genetic mutations. In turn, relying on PGD or seeking gamete donors decreases the risk of having a child with mutations that could lead to genetic diseases. Both procreative behaviours (i.e. procreating or refraining from procreating during the Zika virus epidemic and procreating ‘naturally’ or with technological aid) are protected by reproductive freedom, but they play a role in shaping the structure of the population. This, in turn, can affect the lives of other people. Allen Buchanan (1996, 2011) provides an example that clarifies how the structure of the population, namely *the kind of people that are brought into existence*, has far-reaching effects on both these people and on third parties. The author defines ‘dominant cooperative framework’ the “dominant institutional infrastructure for productive interaction” (Buchanan 1996, p. 40). He argues that choosing which dominant cooperative framework to employ will likely end up favouring the legitimate interests of one group over those of another (Buchanan 2011). Such decision indeed entails determining who will be able to participate in the cooperative interaction and who will not.^5^ To illustrate this point, Buchanan devises the ‘card game analogy’. In this analogy, a group of people gathers to play a card game. While half of these people are able and want to play a complex game such as Bridge, the other half wants to and is only able to play a simple game called Go Fish. If the group decides to play Bridge, then the game will be spoiled as half of the participants would not be able to play effectively. If, instead, the group settles on playing Go Fish, then all the participants will be able to play the game effectively, but those who wanted to play Bridge would have to disregard their interest in playing a more complex game. According to Buchanan, these scenarios illustrate a potential conflict of legitimate interests between a group’s interest in inclusion and another group’s interest in the effective functioning of the cooperative framework. This is relevant for the present discussion. The structure of the population plays a role in determining the kind of cooperative framework that needs to be employed to, on the one hand, favour inclusion, and on the other, favour effective functioning. In turn, the choice of the framework affects both the lives of the procreators and of others.

Naturally and humanly constituted environments of a certain geographical region influence how children born as a result of procreative decisions contribute in different ways to the society they live in. For instance, children born in a region affected by extreme climatic conditions or by violent conflict (say Syria since 2010), as opposed to born in a region with favourable climatic conditions and functioning institutions (say the Silicon Valley in the same period of time) will be able to participate in different ways in the life of the society where they are born and raised, affecting in turn the lives of others born and raised in that society. In Lorenzo Del Savio et al. (2018), we refer to ‘cooperative infrastructures’ as “any material or immaterial technology that contributes to people’s ability to produce human goods through their interaction with others” (Del Savio et al. 2018). The humanly and naturally constituted environments where one is born determine to a large extent the type and quality of the cooperative infrastructures that one has access to, which in turn determine again, to a large extent, the type and quality of the contribution to human goods that one is capable of making (Del Savio et al. 2018).

These are just a few examples of how the effects of procreative decisions, albeit protected by reproductive freedom, have far-reaching effects on third parties and on their legitimate interests. They are also examples of how an individual-centred framework to discuss the ethics of procreation fails to account for such effects. It may seem puzzling that having one or more children or that having a child during the Zika virus epidemic has tangible effects on the overall population. It is true that such decisions, taken separately, do not have significant effects. Despite this, as Dan Brock (2005) argues:

The effect of many individual decisions, themselves each rational and justified as individual choices, may be collectively undesirable [or desirable] for a group or society. ([emphasis added] Brock 2005, p. 378)

Reproductive freedom and the individual-centred framework that this principle rests upon are limited in scope. They fail to include these far-reaching effects on third parties in normative debates on procreation and do not allow for trade-offs in the event of conflicts of legitimate interests. Hence, the question that the rest of this article focuses on is whether there are alternative frameworks to discuss the ethics of procreation that are better suited to ground such normative debates. To address this question, I present and evaluate past and present proposals to reflect on the ethics of procreation in ways that consider such far-reaching and cumulative effects. Concerning the past, I argue that twentieth century eugenics provides an example of conceptualising procreation broadly and an important lesson as to what could go wrong with this approach.^6^ Concerning present proposals to conceive procreation more broadly, I show that an increasing number of authors have begun to advocate for some kind of ‘population engineering’, namely what Colin Hickey and co-authors define as “the intentional manipulation of the size and structure of human populations” (Hickey et al. 2016, p. 845).

## A broader framework: lessons from the past

Central to twentieth century eugenic ideas and movements was a concern for the effects of procreation on the socio-political and economic scaffolding of society. Eugenic-inspired population engineering programmes were constituted by a heterogeneous range of practices, policies, ideologies, movements and thinkers (Adams 1990; Bashford and Levine 2010; Connelly 2008; Meloni 2016; Paul 1984). Eugenic practices included negative interventions such as forced sterilisations of people of colour and of the so-called ‘feeble-minded’ in the U.S. (Kevles 1985; Roberts 1997) and Scandinavian countries (Broberg and Roll-Hansen 2005); mass killings of disabled people such as those committed during the Nazi Aktion T4 programme (Adams 1990; Buchanan et al. 2001); immigration policies aimed at selectively accepting immigrants depending on their geographic and racial origins (Kevles 1985); feminist advocacy for free distribution of birth control domestically (Roberts 1997) and internationally (Connelly 2008; Murphy 2017); and socialist-parties’ attempts to re-organise the welfare state by limiting certain groups’ procreative decisions (Koch 2004; Paul 1984). Part of the history of eugenics were also positive interventions aimed at favouring the birth of strong and healthy individuals, such as the American fitter family contests (Lombardo 2008), ante-natal clinics, school inspection services and free school meals (Porter 2005). In addition to this variation between negative and positive interventions, a diverse range of theories of heredity, with elements of both Lamarkism and Mendelism (Gyngell and Selgelid 2016), as well as of both ‘soft’ and ‘hard’ theories were called into justify eugenic principles and policies (Meloni 2016). Despite this heterogeneity in terms of measures, policies and practices, the idea of changing the size and structure of the population in ways that would increase economic production (Murphy 2017), relieve poverty (Roberts 1997) and favour the breading of a better human stock (Buchanan et al. 2001) was a shared feature of these differing endeavours. During the long history of eugenics, procreation was thought to be a matter of concern for the state rather than something concerning citizens’ private sphere and it was organised to produce aggregate benefits. Various governments around the globe considered legitimate the exercise of control over the bodies of women, immigrants, ethnic minorities, disabled and poor people in order to favour what was believed to be the overall improvement of the gene pool (Connelly 2008).

### What went wrong

What the most despicable features of eugenic ideologies and the programmes enacted in their name were is matter of controversy within normative debates on assisted reproduction (Bashford 2010; Cavaliere 2018). While some features of eugenics such as forced sterilisations are universally condemned, there is no agreement among authors concerning other features. One of the main points of controversy rests on the rightness or wrongness of the aims of eugenics vis-à-vis the means employed to pursue these aims. Eugenics’ quest for genetic improvement is considered by some (see for instance: Agar 2008; Wilkinson 2010) an acceptable aim that was enacted in wrongful and coercive ways. For others, eugenics’ drive towards perfection is intrinsically, i.e. regardless of the means employed, a misguided and wrongful enterprise^7^ (see for instance: Sandel 2007).

In their ‘autopsy of eugenics’, Buchanan et al. (2001) consider five theses to address the question of why eugenics was wrong.^8^ They discard the hypothesis that the wrongness of eugenic programmes rests on their collective rather than individual-centred focus and maintain that “the social goal is not automatically suspect” (Buchanan et al. 2001, p. 55). Rather, they contend that the problem with eugenic programmes is a matter of justice in that they failed to fairly distribute the burdens and benefits of the control exercised over procreation:

The eugenics movements of 1870-1950 insisted – wrongly, as it turned out – that humankind faced a grave threat (degeneration) and stood to gain a large benefit (more able, fit people) if humans would submit to the kind of breeding programs that had been used to improve plants and livestock. *But who would benefit and at whose expenses?* […] The ‘underclass’ is simultaneously the group of people whose genes were not wanted and the people who, through involuntary sexual segregation, stigmatization and denigration, sterilization, and even murder, paid the price. ([emphasis added] Buchanan et al. 2001, p. 52)

Population engineering programmes of the past were designed in such ways that the burdens would systematically fall on certain groups, such as ethnic minorities, disabled people, poor people and immigrants, while the benefits of these programmes would be enjoyed mostly by the ‘fit’, the rich and educated, many of whom were white. Epistemic and political problems were at the heart of this unjust distribution of burdens and benefits, which should be considered within any contemporary attempt to discuss the normative effects of procreative decisions whilst taking into account the interests of people other than the procreators.

These epistemic and political problems can be traced back to how beliefs on the differential economic worth of people for society translated into beliefs of the differential *moral* worth of said people (Murphy 2017). Past population engineering programmes, aimed at enhancing the GDP (Murphy 2017) or at relieving poverty among black minorities in developed countries (Roberts 1997), disproportionally targeted and affected people from the lower classes of developed countries and people living in developing countries. Such beliefs found justification in a view of heredity that Maurizio Meloni (2016) defines as ‘radical biologism’. Manifestations of pauperism, disability and precariousness were linked to biological characteristics and hence considered heritable. These epistemically flawed beliefs served as a source of inspiration for eugenic policies and, at the same time, resonated with extant racist, sexist and ableist political beliefs (Meloni 2016). That epistemically and politically troubling beliefs can give rise to ethically troubling strategies for intervening in procreation is one of the most important lessons to be learned from past population engineering programmes.

## A broader framework: present proposals

Proposals to reflect on procreation in ways that take into account the interests of people other than the procreators are also discussed by contemporary advocates of population engineering. These authors make two types of claims: Malthusian-inspired claims regarding the size of the population (Cafaro 2012; Das Gupta 2014; Hickey et al. 2016; Rieder 2016; Young 2001) and eugenics-inspired claims regarding the structure of the population (Anomaly 2014, 2018; Brock 2005). The first group focuses on the toll of bringing new persons into the world on resources and the environment. They maintain that there are good moral reasons to favour adoption instead of relying on costly assisted reproductive technologies (Overall 2012; Rulli 2016b), to refrain from having more than one child (Rieder 2016), to pursue alternative ways to ‘make kin’ instead of procreating (Haraway 2015) and to forgo or limit procreative aspirations altogether (Cafaro 2012; Conly 2015; Das Gupta 2014; Rieder 2016; Young 2001). The second group focuses instead on population structure and on the costs of unconstrained procreation for current and future generations. Their view is that the problem with an unconstrained procreative behaviour is not ‘too many people’, but too many of a certain kind of people (Anomaly 2014, 2018; Brock 2005). What unites the claims of authors concerned with the size of the population and authors concerned with its structure, is that they question whether an individual-centred framework is appropriate to address normative challenges raised by procreation. To counter the shortcomings of this framework, they frame their proposals in ways that consider the cumulative effects of individual procreative decisions on third parties. These authors also broadly agree that the interests of existing and future people may constitute a *pro tanto* reason in favour of interfering with prospective parents’ reproductive freedom (Anomaly 2014, 2018; Brock 2005; Rieder 2016; Rulli 2016b).

For instance, according to Jonathan Anomaly (2014, 2018), bearing and raising children has “far-reaching effects on the genetic composition, cultural trajectory, and general welfare of future people” (Anomaly 2014, p. 172). Due to these far-reaching effects, procreation needs to be organised in ways which are beneficial to both future people themselves and those around them. One way of doing so would be to influence the structure of the population by favouring the transmission of traits such as creativity, humour, productivity, intelligence and compassion, which are beneficial both to those who have these traits and for others. Adopting an impartial moral standpoint leads to the conclusion that it is better to bring into the world people who will have good lives and whose lives can contribute to the well-being of others (Anomaly 2014). This means that, all things being equal, the birth of children who have traits which are both beneficial to them and to the community of people around them should be favoured. Anomaly grants that more people may translate into more producers, more welfare and a larger work force to support an ageing population, but stresses that people are not equally productive and that “some represent a net cost to their society, or to the world” (Anomaly 2014, p. 176).

While Anomaly is concerned with the structure of the population, other authors are concerned with its size. These authors focus on the different strategies which could be devised to mitigate the negative effects of climate change and to reduce anthropogenic greenhouse gas emissions. Travis Rieder (2016) and others (Das Gupta 2014; Hickey et al. 2016; Murtaugh and Schlax 2009) contend that the most effective way to reduce such emissions is to decrease the size of the population by changing people’s procreative behaviours, as procreation plays an important role with respect of the quantity of these emissions (Harte 2007; Murtaugh and Schlax 2009; Nolt 2011). The effects of climate change on people’s well-being call, in their view, for *pro tanto* moral reasons to refrain from procreation or, at least, for reducing the number of children being born. For instance, Rieder (2016) argues that people have “procreation-limiting duties” (Rieder 2016, p. 9) as:

There are too many people on earth, together emitting too much GHG much too quickly. […] The public health crisis of overpopulation leads to the intuitive conclusion that morality might demand of each of us that we do not contribute to such a crisis. (Rieder 2016, p. 10)

Authors in favour of carrying out population engineering advocate for measures that seek to address normative challenges raised by the far-reaching effects of individual procreative decisions. At the same time, they are cautious as to the potential negative externalities of curtailing people’s freedom. They adopt different strategies to address the tension between the interests (and the freedom) of individual procreators and the interests of other people. Rieder (2016), for instance, argues that there is more to morality than what is within one’s rights. He contends that acting morally entails reducing one’s own family size even if this is at odds with one’s own rights. Anomaly (2014) instead focuses on reasons to exercise caution in “moving from social norms that nudge people to make socially beneficial reproductive choices, to using state institutions that shape reproductive choices” (Anomaly 2014, p. 182). These reasons for caution are: the lack of adequate genetic knowledge; the value of reproductive freedom; and that agents carrying out population engineering may “possess imperfect information” and “face perverse incentives” (Anomaly 2014, p. 182).

The knowledge of how genes influence behavioural traits such as empathy and intelligence (or even aesthetic traits such as eye colour or height) and the capacity to edit genes to favour the expression of these traits are in their infancy to say the least. Despite this, I would argue that the current lack of knowledge may not represent per se an insurmountable challenge to carrying out population engineering programmes. It may also not represent, as it were, an insurmountable (ethical) argument against these programmes. Studies on the hereditability of IQ date back to the beginning of the twentieth century and some progress has been made (for a review, see for instance: Ritchie 2015). Moreover, if it becomes clear that population engineering and Anomaly’s aims of improving the structure of the population can be achieved not through prenatal/pre-conception genetic interventions, but thanks to controlled epigenetic influence, education, welfare provisions and other post-natal measures, then the question of the desirability of population engineering programmes will remain. In other words, the first reason for caution identified by Anomaly (2014) is a contingent matter. Similarly, with respect to the second reason for caution, it is important to consider that even liberal defences of reproductive freedom allow some degree of interference from third parties. What matters is hence establishing whether the harms engendered by what reproductive freedom protects warrant some kind of restriction on people’s procreative decisions. This needs to be discussed while bearing in mind a third reason for caution identified by Anomaly (2014), namely the risk that third parties may “possess imperfect information” and “face perverse incentives” (Anomaly 2014, p. 182). It is to this third reason that I now turn.

### What could go wrong

As I have argued above, an important lesson of past proposals to rethink procreation is that epistemically and politically troubling beliefs can give rise to ethically troubling strategies for intervening in procreation. The problem of these past proposals is not the adoption of a broader framework to reflect on and organise procreation. Rather, their troubling component rests on how trade-offs between competing interests were made and burdens and benefits were distributed. In this section, I argue that this lesson is also relevant for contemporary defences of population engineering programmes.

I identify two main shortcomings of present population engineering programmes and proposals: acquiring reliable data on who should come into existence and assessing this data. Regarding proposals aimed at tackling the structure of the population, the first shortcoming concerns the feasibility of acquiring data on the type of people who could be reliably said to contribute to overall increases in the well-being of future people. Anomaly (2014) argues that the best suited to become parents are those with “favorable genetic endowment” and “the means to provide a rich social environment for their children” (Anomaly 2014, p. 174), as both characteristics seem to predict the birth of people whose lives have value both for themselves and for others. However, what counts as favourable genetic endowment and as a rich social environment is a complex notion: whether a given genetic endowment really turns out to be favourable often depends also on people’s social environments. In this sense, the assessment of what counts as favourable genetic endowment cannot be separated from the assessment of what counts as a rich social environment. Not only are these conditions often context-dependent, they are also normatively loaded as what counts as ‘favourable’ and ‘rich’ presupposes the adoption of a certain normative framework as a reference. In other words, in an assessment of whether something is rich or favourable, an impartial moral standpoint will not do. Different groups of people are likely to come up with different assessments of what counts as valuable and competing interests are likely to play a role in these assessments.

This brings me to the second shortcoming. If I am right about the first, there will be competing assessments of what counts as valuable, and therefore different answers to the question of what type of people should be allowed to come into existence. Hence, a reliable mechanism to acquire empirical data that can assist in the selection of the best answers to ground future policies becomes necessary. Acquiring this data seems again normatively loaded and complex: this data could reflect our current ‘status quo biases’ (Bostrom and Ord 2006), racist and discriminatory attitudes (Roberts 1997, 2015) and short-sighted or partial conceptions of valuable lives (Garland-Thomson 2012; Mackenzie and Scully 2007). There are existing studies that document IQ hereditability and a correlation between high IQ and low fertility (Meisenberg 2009). Despite this, delving into the history of research on the mechanisms of human heredity allows to appreciate that a complicated interplay between epistemic and political forces exists: between the quest for knowledge and interventions acting upon that knowledge (Meloni 2016; Roberts 2015). The type of questions asked, the hypotheses formulated, the data collected, and the inferences drawn are all likely to be influenced by existing political views and beliefs (Kitcher 2001). In addition, it is unclear who should decide what data is to be taken into consideration for institutional design and whose normative framework should be used as a reference. The problem, then, is not (or not only) about ‘imperfect information’ and ‘perverse incentives’ possessed by the state, but rather about the difficulty of having reliable mechanisms to assess this information both in the context of state interventions and of shaping social norms.

Regarding the size of the population, it may seem that the proposals outlined above might be less problematic and that the challenges discussed might not apply. Economic growth, increased levels of welfare, empowering women, better educational provision and institutions are all viable strategies to reduce fertility rates. Despite this, the one-size-fits-all model to reduce the size of the population seems to be ill-conceived if the aim is to reduce, for example, climate change hazards (as noted for instance by Haraway 2015; Rieder 2016). Not all people contribute equally to the worsening of climate change and not all people are in an equal position to produce new resources to minimise these negative effects. Hence, it seems reasonable to assume that it would be self-defeating to implement measures which seek to reduce everyone’s birth rates. Population engineering programmes aimed at reducing the size of the population cannot be easily disentangled from population engineering programmes aimed at influencing the structure of the population. The two (i.e. size and structure) cannot be completely separated today, and they were not completely separated in the past^9^ (Bashford 2014; Connelly 2008; Klausen and Bashford 2010). Contemporary attempts to reduce the size of the population are likely to incur the very same shortcomings identified above, as competing interests are likely to influence people’s assessments of what counts as valuable (i.e. who should procreate and how much) and the decision of who gets to decide in these matters influences which interests will be given priority.

## Addressing asymmetries

What then should guide normative debates on procreative decisions? What interests should be taken into consideration and what kind of principles and frameworks are suited to protect them? I have argued that one of the major shortcomings of an individual-centred framework is that it conceives procreation as a private matter and it fails to account for the effects of procreative decisions on third parties and their legitimate interests. Insofar as it rests upon this framework, reproductive freedom as a moral principle to guide the normative debate on procreative decisions and as a standard to make trade-offs between competing interests is limited in scope. For this reason, past and present population engineering programmes may be shaped upon normative considerations that are better suited to discuss the ethics of procreation and to consider the interests of people other than the procreators. Despite these merits, past population engineering programmes ended up unfairly distributing the burdens and benefits of carrying out such programmes. In my view, present proposals risk incurring in similar problems, as lingering discriminatory and partial attitudes may inform the structural design of population engineering programmes. Hence, I argue that any attempt to reflect on procreation more broadly would first have to address what Philip Kitcher (2001) refers to as political and epistemic asymmetries. According to Kitcher (2001), political asymmetry occurs if (a) empirical data that supports certain lingering sexist or racist beliefs leads to reverting to a situation in which these beliefs were widespread, while empirical data that contradicts these lingering beliefs does not lead to a further eradication of these beliefs; and if (b) empirical data that supports certain lingering sexist or racist beliefs leads to the worsening of certain racial groups’ or of women’s lives, while empirical data that contradicts these lingering beliefs does not lead to notable improvements for these groups (Kitcher 2001, p. 97). Epistemic asymmetry occurs instead when certain studies and conclusions, theories and data, despite being assigned low reliability, will be taken more seriously than they should be (considering the low reliability) if they resonate with widespread racist and sexist beliefs. Population engineering programmes may end up constraining or influencing people’s procreative decisions and interfering with their freedom. Due to the relevance of procreation for people’s well-being and due to the tainted history of attempts to engineer the population, it is necessary to develop reliable strategies to make sure that political and epistemic asymmetries do not persist. In cases where stakes are so high, “standards of evidence must go up” (Kitcher 2001, p. 96). Unless epistemic and political asymmetries are properly addressed, it seems that reflecting more broadly on procreation and considering the effects of procreative decisions on third parties is not without risk.

These considerations confront those who discuss the ethics of procreative decisions with a dilemma: the first horn of the dilemma entails that a principle that protects procreators’ interests such as reproductive freedom is the most suited against potential violations of, and interference with, people’s procreative projects. The second instead entails that the effects of procreative decisions on third parties’ relevant interests are not sufficiently taken into account if normative debates on procreative decisions continue to be grounded in an individual-centred framework. Until epistemic and political asymmetries are properly addressed and the risks of unfairly distributed burdens and benefits of population engineering programmes are minimised, I propose a way around this dilemma. This would entail defending people’s reproductive freedom as a theoretically imperfect but ad interim instrumentally necessary tool. The protection that reproductive freedom grants to people’s interests in making decisions about their procreative projects autonomously is theoretically flawed as it rests upon a narrow conception of the effects of procreative decisions. It is also normatively problematic in that it protects procreators’ interests whilst not allowing to make trade-offs with other people’s potentially competing interests. Hence, reproductive freedom should not be championed as what best protects people’s interests in well-being and autonomy. Rather, it should be championed as what at best (and ad interim) protects potentially vulnerable groups from harmful interferences. Within policy making and programmes aimed at regulating new reproductive technologies, screening technologies, terminations of pregnancies and so forth, reproductive freedom should still guide decision-making, until epistemic and political asymmetries are properly addressed. At the same time, considering the far-reaching effects of procreative decisions could enable to normatively appraise such effects on third parties and their interests.

## Conclusion

In this article, I have argued that while reproductive freedom presents several shortcomings that have to do with the individual-centred framework that this principle rests upon. I have suggested that both twentieth century eugenics and contemporary proposals to carry out population engineering programmes are grounded in broader frameworks that allow to include reflections on the normative implications of procreative decisions. Despite this, both past and present proposals risk incurring in an unfair distribution of the burdens and benefits of population engineering programmes, unless epistemic and political asymmetries are addressed. For this reason, I have suggested that reproductive freedom should be defended as a theoretically imperfect but ad interim instrumentally necessary tool.

## Footnotes

1 I return to some of these critiques in “A broader framework: present proposals” section, where I limit my analysis to those motivated by a concern for the well-being and interests of others (i.e. others than the procreators and their close networks).

2 I am indebted to an anonymous reviewer for bringing these issues to my attention.

3 In this paper, I follow Hickey et al.’s (2016) distinction between size and structure and adopt these two categories in my analysis of the effects on third parties of procreative decisions. While what Hickey et al. (2016) mean by population ‘size’ strikes me as intuitive, when I discuss population ‘structure’ I am referring to the composition of the population: namely to the *type* of people who collectively shape the structure of the population.

4 It must be noted that here I remain neutral as to whether these changes are desirable or not as this depends on a number of variables (such as for instance how these changes are designed and implemented and to serve what interests). What matters for the present discussion is that population’s size affects the lives of other people than the individual procreators and their close network.

5 In Buchanan’s (1996) words: “choosing the dominant cooperative scheme means choosing who will and who will not be disabled” (Buchanan 1996, p. 40).

6 In discussing the history of eugenics, I follow the tradition of authors who engage with such history and with eugenics’ underpinning ideologies, practices and policies while resisting outright condemnations (see for instance: Agar 2008; Buchanan et al. 2001; Glover 2006; Selgelid 2014; Wilkinson 2010).

7 For a comprehensive review and assessment of these claims, see Giulia Cavaliere (2018).

8 The theses are: ‘replacement, not therapy’; ‘value pluralism’; violations of reproductive freedom’; ‘statism’ and ‘justice’ (Buchanan et al. 2001; see also: Wikler 1999).

9 As argued by Alison Bashford: “[i]f population growth was to be reduced, the eugenic question was derivative: which kind of person might be reproductively restrained or encouraged?” (Bashford 2014, p. 242).
